# Two monogenic disorders masquerading as one: severe congenital neutropenia with monocytosis and non-syndromic sensorineural hearing loss

**DOI:** 10.1186/s12881-020-0971-z

**Published:** 2020-02-17

**Authors:** Parvathy Venugopal, Lucia Gagliardi, Cecily Forsyth, Jinghua Feng, Kerry Phillips, Milena Babic, Nicola K. Poplawski, Hugh Young Rienhoff, Andreas W. Schreiber, Christopher N. Hahn, Anna L. Brown, Hamish S. Scott

**Affiliations:** 1 Genetics and Molecular Pathology, SA Pathology, Adelaide, Australia; 2 Centre for Cancer Biology, an alliance between SA Pathology and the University of South Australia, Adelaide, Australia; 3 School of Medicine, University of Adelaide, Adelaide, Australia; 4 Endocrine and Diabetes Unit, The Queen Elizabeth Hospital, Woodville South, Australia; 5 Endocrine and Metabolic Unit, Royal Adelaide Hospital, Adelaide, Australia; 6 Jarrett Street Specialist Centre, Gosford, Australia; 7 Australian Cancer Research Foundation Cancer Genomics Facility, Centre for Cancer Biology, SA Pathology, PO Box 14, Rundle Mall, Adelaide, South Australia 5000 Australia; 8 School of Pharmacy and Medical Sciences, Division of Health Sciences, University of South Australia, Adelaide, Australia; 9 Adult Genetics Unit, Royal Adelaide Hospital, Adelaide, Australia; 10 Imago BioSciences, Inc., San Francisco, California USA; 11 School of Biological Sciences, University of Adelaide, Adelaide, Australia

**Keywords:** Neutropenia, Congenital neutropenia, Leukemia predisposition, Polygenic inheritance, Hearing loss

## Abstract

**Background:**

We report a large family with four successive generations, presenting with a complex phenotype of severe congenital neutropenia (SCN), partially penetrant monocytosis, and hearing loss of varying severity.

**Methods:**

We performed whole exome sequencing to identify the causative variants. Sanger sequencing was used to perform segregation analyses on remaining family members.

**Results:**

We identified and classified a pathogenic GFI1 variant and a likely pathogenic variant in MYO6 which together explain the complex phenotypes seen in this family.

**Conclusions:**

We present a case illustrating the benefits of a broad screening approach that allows identification of oligogenic determinants of complex human phenotypes which may have been missed if the screening was limited to a targeted gene panel with the assumption of a syndromic disorder. This is important for correct genetic diagnosis of families and disentangling the range and severity of phenotypes associated with high impact variants.

## Background

Severe congenital neutropenia (SCN) was first described by Kostmann in 1956 in 14 individuals from 9 consanguineous families [1]. It is usually diagnosed in early childhood and is characterized by chronic neutropenia, susceptibility to bacterial infections and associated with a predisposition to myelodysplastic syndrome (MDS) or acute myeloid leukemia (AML). SCN is genetically heterogeneous with autosomal recessive, autosomal dominant (AD) and X-linked forms described as well as de novo cases [2]. It may also occur as a part of a syndrome with other developmental defects (e.g. Shwachman-Diamond Syndrome) [3]. We describe a family with a complex phenotype of SCN and hearing loss of varying severity.

The genetic basis of SCN is well described and involves mutations in a number of different genes (Supplemental Table S2). Pathogenic variants in *ELANE*, the gene encoding neutrophil elastase, are the most common cause of AD SCN. Growth factor independent 1 transcriptional repressor (*GFI1*) germline variants have been reported in four patients with neutropenia; inheritance patterns were consistent with an AD mode of inheritance [4]. *HAX1* variants underlie some autosomal recessive forms and were found to be the underlying cause of SCN in the families originally described by Kostmann [5]. It has been suggested that these variants impair neutrophil maturation via defective CSF3R signaling as the number of G-CSF receptors on myeloid precursors of SCN patients is elevated and majority of SCN patients benefit from the administration of pharmacological doses of granulocyte colony stimulating factor (G-CSF) [6].

Depending on the causative genetic lesion, neutropenia can sometimes present with extra-haematopoietic abnormalities such as pancreatic exocrine insufficiency (*SBDS, ELF1*) and deafness (*GATA2*). We previously reported heritable variants in *GATA2* as predisposing to familial MDS and AML [7]. Since then, a high incidence of *GATA2* variants has been found in patients with mild neutropenia who evolve to develop MDS and AML [8]. Interestingly, sensorineural hearing loss and monocytopenia are other manifestations associated with haploinsufficiency of *GATA2*. Here, we report a large family with multiple generations affected by neutropenia and hearing loss.

## Methods

### Patient samples

Blood and hair samples were obtained with consent from members of the family as part of the Australia Familial Haematological Cancer Study (AFHCS). gDNA was isolated using the QIAamp DNA Mini Kit (Qiagen).

### Whole exome sequencing (WES)

We performed WES (SeqCap EZ MedExome, Roche NimbleGen) on two individuals (III-1 proband and IV-1). Variant annotation was performed through our ACRF Cancer Genomics Facility custom pipeline, which takes into consideration pathogenicity/oncogenicity predictions (CADD> 10, Polyphen 2, SIFT, Mutation Taster, GERP > 2, COSMIC parameters including specific-mutation and gene frequency), population minor allele frequencies (1000 GP, ESP, gnomAD), OMIM, and Gene Ontology. All candidate variants were manually curated to remove errors. The variants were interrogated for known SCN genes (Table S2) and non-syndromic hearing loss (Table S3).

### Sanger sequencing

Sanger sequencing was performed on available samples for confirmation of *GFI1* and *MYO6* variants in family members to perform segregation analysis.

## Results

**Fig. 1:**
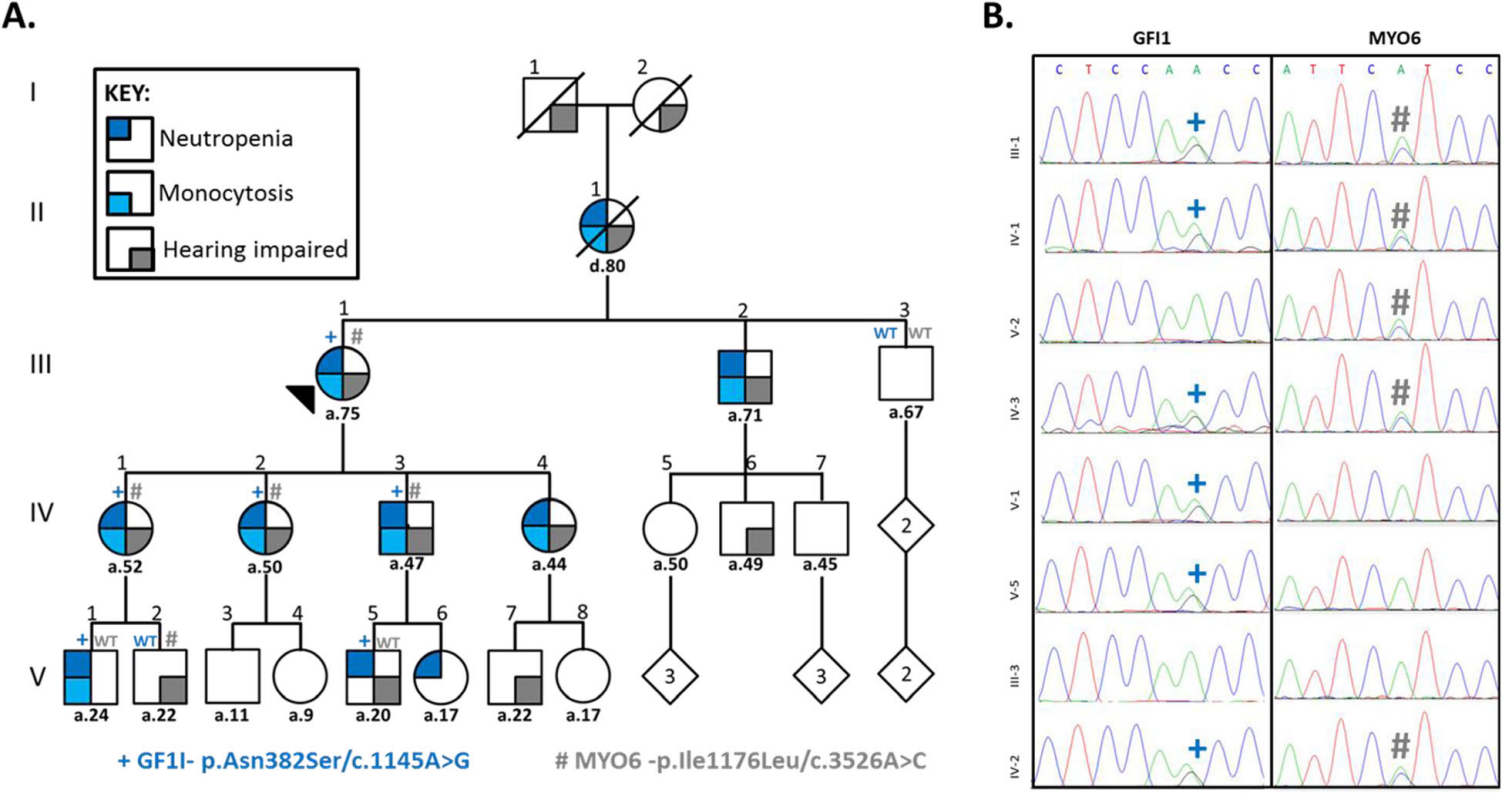
Family with inherited neutropaenia, monocytosis and hearing impairment associated with mutations in GFI1 and MYO6. Pedigree, phenotypes and mutation status are indicated as per the key provided (**a**). Causative heterozygous mutations in GFI1 (p.N382S/c.1145A > G) and MYO6 (p.I1176L/c.3526A > C) were identified by whole exome sequencing performed on III-1 and IV-1. Sanger sequencing on available samples from consenting individuals was used for segregation analysis and confirmation of variants in individuals denoted by ‘+’ and ‘#’, respectively (**b**)

We identified a five-generation kindred with four successive generations affected by congenital neutropenia (10 individuals; eight also had monocytosis – available blood counts in Table S1) and five generations affected by hearing loss of varying severity (13 individuals) (Fig. 1a, Table 1). Sanger sequencing of *GATA2* was performed due to partial overlap in the phenotypes observed within the family, but no pathogenic coding variants were found. We therefore performed whole exome sequencing on III-1 and IV-1(see Methods). The variants were interrogated for known SCN genes (Supplemental Table S2). We identified a previously reported variant underlying congenital neutropenia in *GFI1* (NM_005263.5, c.1145A > G/p.Asn382Ser, N382S) (Fig. 1b, left panel) [4]. The N382S variant segregated with neutropenia in 6 consenting family members who were tested (Table 1) including V-5 who reported having had low white cells counts (blood reports unavailable). With the addition of these 6 cases to the 3 previously reported individuals, this variant can now be classified as a pathogenic variant, as per ACMG variant classification guidelines (Supplemental Table S4) [9].

**Table 1:** Genotypes and phenotypes of various members in the family

Individual	GFI1	Blood	MYO6	Hearing
III-1	N382S (het)	**Neutropenia, monocytosis**	I1176L (het)	**Impaired**
III-3	WT	Normal	WT	Normal
IV-1	N382S (het)	**Neutropenia, monocytosis**	I1176L (het)	**Impaired**
IV-2	N382S (het)	**Neutropenia, monocytosis**	I1176L (het)	**Impaired**
IV-3	N382S (het)	**Neutropenia**	I1176L (het)	**Impaired**
V-1	N382S (het)	**Neutropenia, monocytosis**	WT	Normal
V-2	WT	Normal	I1176L (het)	**Very slight hearing loss**
V-5	N382S (het)	Has had low white cell count^a^	WT	**Slight hearing loss**
I-1	Unknown	Unknown	Unknown	**Impaired**
I-2	Unknown	Unknown	Unknown	**Impaired in later years**
II-1	Unknown	**Neutropenia, monocytosis**	Unknown	**Impaired**
III-2	Unknown	**Neutropenia, monocytosis**	Unknown	**Impaired**
IV-4	Unknown	**Neutropenia**	Unknown	**Impaired**
IV-5	Unknown	Normal	Unknown	Normal
IV-6	Unknown	Unknown	Unknown	**Impaired**
IV-7	Unknown	Normal	Unknown	Normal
V-3	Unknown	Normal	Unknown	Normal
V-4	Unknown	Normal	Unknown	Normal
V-6	Unknown	Has had low white cell count^a^	Unknown	Normal
V-7	Unknown	Unknown	Unknown	**Slight hearing loss**
V-8	Unknown	Unknown	Unknown	Normal

*Abbreviations*: *WT* Wildtype, *het* Heterozygous

^a^reported in patient questionnaires/interview – no accompanying blood reports available

As *GFI1* variants have not previously been associated with hearing loss, we explored additional genetic causes for the hearing loss phenotype observed in this family. Over 100 genes have been reported to underlie hereditary hearing loss. Analysis of the data for variants in genes associated with non-syndromic hearing loss (Supplemental Table S3), identified a novel single nucleotide variant in *MYO6* (NM_004999.4, c.3526A > C/p.Ile1176Leu, I1176L) (Fig. 1b, right panel), a gene that has previously been associated with AD hearing loss [10]. The novel MYO6 I1176L variant segregated with hearing loss in all but one individual (V-5) who displayed clinically unconfirmed mild hearing loss (Fig. 1a). Supplemental Table S4 summarizes variant annotation and classification.

## Discussion

Congenital neutropenia and monocytosis due to *GFI1* variants was first reported in 2003, following the observation that *GFI1*-deficient mice were unexpectedly neutropenic [4]. *GFI1* encodes a zinc finger transcriptional repressor oncoprotein. The N382S variant occurs in a highly evolutionarily conserved region of the GFI1 protein (Supplemental Figure S1). In vitro studies demonstrated that the variant acted in a dominant negative manner, abolishing DNA binding and hence repressor activity of the protein [4]. Interestingly, GFI1 recruits to chromatin the enzyme lysine-specific demethylase-1 (LSD1); pharmacologic inhibition of LSD1 or genetic knock-down of LSD1 skews granulocyte-monocyte progenitor differentiation resulting in neutropenia and monocytosis as seen in this family [10]. LSD1 is currently a target for the treatment of AML (NCT02842827).

SCN can be a pre-leukemic syndrome with evolution to leukemia recognized in patients with *ELANE* and *HAX1* variants, as well as X-linked neutropenia (*WAS)*. Patients with SCN reportedly have a 21% cumulative incidence of myeloid malignancy after 10 years with the risk of leukemia being higher in patients requiring high doses of G-CSF, and is associated with acquired mutations in *CSF3R* and *RUNX1* [11].

To date, there have been no reports of MDS or AML in individuals with GFI1 variants alone (3 with N382S, 1 with K403R, 1 with R412*) [4] with the exception of three individuals who carried germline variants in both *GFI1* and *ELANE* [12]. Our family adds another 6 cases of confirmed *GFI1* variant carriers (aged 20–75 years) and 2 likely carriers (over 71 years) without progression to myeloid malignancy, suggesting that the *GFI1* variant alone does not confer a high risk of leukemia development.

Variants in myosin genes are known to be involved in several types of syndromic and non-syndromic hearing loss. Variants in myosin VI (*MYO6*) have been identified in AD and recessive hearing loss [10, 13]. The primary evidence for the association of myosin VI with the hearing process is based on the Snell’s waltzer mice, that exhibit deafness [14]. The myosin VI protein is highly expressed at the base of stereocilia rootlets and in the pericuticular necklace of the inner and outer hair cells of the organ of Corti. MYO6 is required for the proper maturation of inner hair cell ribbon synapses and it has been shown to interact with DFNB9 (responsible for a recessive form of deafness) via the globular domain [15]. The I1176L variant is surrounded by a block of conserved amino acids and resides within a very highly conserved region (Figure S1) showing 91% amino acid sequence identity from p.Asn1165- Lys1285, that encodes the globular domain in the protein [15]. Miyagawa et al. have reported various other variants within this globular domain where the degree of hearing loss ranged from mild to profound.

Intriguingly, *GFI1* has also been reported to be essential for inner ear hair cell differentiation [15, 16]. Hence individual V-5, who has reported slight hearing loss and has tested wildtype for *MYO6*, but carries the *GFI1* variant, is an interesting case in this respect. Another curious observation is that family members with severe hearing impairment carry both variants while those with milder hearing problems only carry either of the variants. Though the *MYO6* variant is most likely responsible for hearing loss in the family, it remains to be established if the *GFI1* variant contributes to the phenotype.

## Conclusions

We have presented a family with a complex phenotype of SCN and hearing loss that can be attributed to AD variants in two genes, *GFI1* causing SCN, and *MYO6* leading to hearing loss. Broader screening may be warranted in cases with complex disease presentations as polygenic inheritance could be missed if testing is restricted to specific gene panels. Although progression to leukemia has not been described to date in the limited number of individuals carrying germline pathogenic GFI1 variants, it remains to be established whether molecular monitoring for acquired variants should be considered as part of a risk management scheme.

## Supplementary information

**Additional file 1: Table S1.** Neutrophil and monocyte counts of individuals where data was available. **Table S2.** Genes known to be mutated in congenital neutropenia. **Table S3.** Genes known to be mutated in non-syndromic hearing loss. **Table S4.** Computational predictions, variant annotation and classification.

**Additional file 2: Figure S1.** Sequence conservation of mutated residues in GFI1 and MYO, across species.

## Data Availability

The datasets generated and/or analysed during the current study is available in the EGA repository (Study ID: EGAS00001004176). These are accessible via the following links: https://www.ebi.ac.uk/ega/studies/EGAS00001004176; https://www.ebi.ac.uk/ega/datasets/EGAD00001005937.

## Abbreviations

AD: Autosomal dominant
AML: Acute myeloid leukemia
G-CSF: Granulocyte colony stimulating factor
LSD-1: Lysine-specific demethylase-1
MDS: Myelodysplastic syndrome
SCN: Severe congenital neutropenia
