# Supplementary material for: Two monogenic disorders masquerading as one: severe congenital neutropenia with monocytosis and non-syndromic sensorineural hearing loss
Source: BMC Med Genet. 2020 Feb 17;21:35. doi: 10.1186/s12881-020-0971-z (PMC7026993; doi:10.1186/s12881-020-0971-z)
Supplement: Supplementary file 1 — Additional file 1: Table S1. Neutrophil and monocyte counts of individuals where data was available. Table S2. Genes known to be mutated in congenital neutropenia. Table S3. Genes known to be mutated in non-syndromic hearing loss. Table S4. Computational predictions, variant annotation and classification. [file 12881_2020_971_MOESM1_ESM.pdf]

Supplementary Tables:

Table S1: Neutrophil and monocyte counts of individuals where data was available. Readings outside the reference range (Ref) have been shown in bold.

Parameter		Ref	II-1	III-2	IV-1	IV-2	IV-3	IV-4	V-1
Neutrophils	$x10^9/L$	2.0-7.5	**0.957**	**0.8**	**0.76**	**0.5**	2.45	**1.1**	**0.7**
Monocytes	$x10^9/L$	0-1.0	**1.122**	**2.1**	**1.68**	**1.6**	**1.72**	**1.3**	**1.5**

Table S2: Genes known to be mutated in congenital neutropenia (Skokowa *et al*. 2017).

**Autosomal Dominant Inheritance**				
ELANE	***GFI1***	*GATA2*	*TCIRG1*	*CXCR4*
CSF3R				
**Autosomal Recessive Inheritance**				
HAX1	*JAGN1*	*G6PC3*	*SLC37A4*	*SBDS*
STK4	*CLPB*	*AP3B1*	*LAMTOR2*	*USB1*
VPS13B	*VPS45*	*CXCR2*	*EIFAK3*	*LYST*
RAB27A	*AK2*	*RMRP*	*TCN2*	*CSF3R*
**X-linked Inheritance**				
WAS	*TAZ*	*CD40LG*		

Table S3: Genes known to be mutated in non-syndromic hearing loss (Shearer *et al.*, 2017)**.**

**Autosomal Dominant Inheritance**				
ACTG1	*CCDC50*	*CD164*	*CEACAM16*	*COCH*
COL11A2	*GSDME*	*DIAPH1*	*DMXL2*	*DSPP*
EYA4	*GJB2*	*GJB3*	*GJB6*	*GRHL2*
HOMER2	*KCNQ4*	*MIR96*	*MCM2*	*MYH14*
MYH9	*MYO1A*	***MYO6***	*MYO7A*	*OSBPL2*
P2RX2	*POU4F3*	*SIX1*	*SLC17A8*	*TBC1D24*
TECTA	*TJP2*	*TMC1*	*WFS1*	
**Autosomal Recessive Inheritance**				
ADCY1	*BDP1*	*BSND*	*CABP2*	*CDC14A*
CDH23 1	*CIB2*	*CLDN14*	*CLIC5*	*COL11A2*
DCDC2	*PJVK*	*ELMOD3*	*EPS8*	*EPS8L2*
ESPN	*ESRRB*	*GIPC3 2*	*GJB2 3*	*GJB6 3*
GPSM2	*GRXCR1*	*GRXCR2*	*HGF*	*ILDR1*
KARS	*LHFPL5*	*LOXHD1*	*LRTOMT*	*MARVELD2*
MET	*MSRB3*	*MYO15A*	*MYO3A*	*MYO6*
MYO7A 5	*NARS2 6*	*OTOG*	*OTOGL*	*OTOA*
OTOF	*PCDH15*	*PNPT1*	*PTPRQ*	*RDX*
RIPOR2	*ROR1 7*	*S1PR2*	*SERPINB6*	*SLC22A4*
SLC26A4	*SLC26A5*	*STRC*	*SYNE4*	*TECTA*
TBC1D24	*TMC1*	*TMEM132E*	*TMIE*	*TMPRSS3*
TPRN	*TRIOBP*	*TSPEAR*	*USH1C 12*	*WBP2*
WHRN				
**X-linked Inheritance**				
PRPS1	*POU3F4*	*SMPX*	*AIFM1*	*COL4A6*

Table S4: Computational predictions, variant annotation and classification.

HGNC Symbol	Position (hg19)	Amino Acid/ Nucleotide Change	CADD	GERP	Population Frequency (gnomAD)	ACMG Classification
**GFI1**	Chr1:92,941,710	p.Asn382Ser/ c.1145A>G NM_005263.5	23.9	4.18	0	Pathogenic
**MYO6**	Chr6:76,623,866	p.Ile1176Leu/ c.3526A>C NM_004999.4	25.3	5.89	3/277220	Likely pathogenic

Supplemental References:

Shearer, A.E., Hildebrand, M.S., Smith, R.J.H. Hereditary Hearing Loss and Deafness Overview. Adam MP, Ardinger HH, Pagon RA, et al., editors. GeneReviews® [Internet]. Seattle (WA): University of Washington, Seattle; 1993-2019. Available from: https://www.ncbi.nlm.nih.gov/books/NBK1434/

Skokowa, J., Dale, D. C., Touw, I. P., Zeidler, C. & Welte, K. (2017). Severe congenital neutropenias. *Nature Reviews Disease Primers* **3**: 17032.
