# Supplementary material for: Two monogenic disorders masquerading as one: severe congenital neutropenia with monocytosis and non-syndromic sensorineural hearing loss
Source: BMC Med Genet. 2020 Feb 17;21:35. doi: 10.1186/s12881-020-0971-z (PMC7026993; doi:10.1186/s12881-020-0971-z)
Supplement: Supplementary file 2 — Additional file 2: Figure S1. Sequence conservation of mutated residues in GFI1 and MYO, across species. [file 12881_2020_971_MOESM2_ESM.pdf]

A.

H. sapiens	340	YPCQYCGKRFHQKSDMKKHTFIHTGEKPHKCQVCGKAFSQSSNLITHSRK	389
P. troglodytes	162	YPCQYCGKRFHQKSDMKKHTFIHTGEKPHKCQVCGKAFSQSSNLITHSRK	211
M. mulatta	340	YPCQYCGKRFHQKSDMKKHTFIHTGEKPHKCQVCGKAFSQSSNLITHSRK	389
C. lupus	340	YPCQYCGKRFHQKSDMKKHTFIHTGEKPHKCQVCGKAFSQSSNLITHSRK	389
B. taurus	338	YPCQYCGKRFHQKSDMKKHTFIHTGEKPHKCQVCGKAFSQSSNLITHSRK	387
M. musculus	341	YPCQYCGKRFHQKSDMKKHTFIHTGEKPHKCQVCGKAFSQSSNLITHSRK	390
R. norvegicus	341	YPCQYCGKRFHQKSDMKKHTFIHTGEKPHKCQVCGKAFSQSSNLITHSRK	390
G. gallus	315	YPCQYCGKRFHQKSDMKKHTFIHTGEKPHKCQVCGKAFSQSSNLITHSRK	364
D. Rerio	314	YPCQYCGKRFHQKSDMKKHTFIHTGEKPHKCQVCGKAFSQSSNLITHSRK	363
X. tropicalis	301	YPCQYCGKRFHQKSDMKKHTFIHTGEKPHKCQVCGKAFSQSSNLITHSRK	350

B.

H. sapiens	1135	TDYDFAPFLNNSPQQNPAAQIPARQREIEMNRQQRFFRIPFIRPADQYKD	1184
P. troglodytes	1135	TDYDFAPFLNNSPQQNPAAQIPARQQEIEMNRQQRFFRIPFIRPADQYKD	1184
M. mulatta	1112	TDY---------AQQNPAAQIPARQQEIEVNRQQRFFRIPFIRPADQYKD	1152
C. lupus	1131	TDYDFAPFLNNSPQQNPASQLPARQQEMEMNRQQRFFRIPFIRPADQYKD	1180
B. taurus	1136	TDYDFAPFLNNSPQQNPAAQLPARQQEIEMNRQQRFFRIPFIRPADQYKD	1185
M. musculus	1112	TDYDFAPFLNNSPQQNPAAQLPARQQEIDMKRQQRFFRIPFIRPADQYKD	1161
R. norvegicus	1106	TDYDFAPFLNNSPQQNPAAQLPARQQEIEMNRQQRFFRIPFIRPADQYKD	1155
G. gallus	1135	TDY---------AQQNPTAQLPMRQQEIEINRQQRYFRIPFIRPMDQYKD	1175
D. rerio	1126	TDY---------AQQNPAPPVPARQQEIAMNRQQRYFRIPFIRPADQYKD	1166
X. tropicalis	1137	TDYDFAPFMNNSPQQNPVVQLPARQQEIEMNRQQRYFRIPFIRPADQYKD	1186

Fig SI: Sequence conservation of mutated residues in *GFI1* and *MYO*, across species. Mutations found in the family in *GFI1* (p.N382S/c.1145A>G) (A) and *MYO6* (p.I1176L/c.3526A>C) (B) are in residues that are highly conserved across species.
